# Molecularly Responsive Aptamer-Functionalized Hydrogel for Continuous Plasmonic Biomonitoring

**DOI:** 10.1021/jacs.5c01718

**Published:** 2025-03-20

**Authors:** Soohyun Park, Alice Gerber, Cátia Santa, Gizem Aktug, Bastian Hengerer, Heather A. Clark, Ulrich Jonas, Jakub Dostalek, Khulan Sergelen

**Affiliations:** † BioMed X Institute, Heidelberg 69120, Germany; ‡ Faculty of Biotechnology, Mannheim University of Applied Sciences, Mannheim 68163, Germany; § FZU-Institute of Physics, Czech Academy of Sciences, Prague 180 00, Czech Republic; ∥ Department of Biophysics, Chemical and Macromolecular Physics, Faculty of Mathematics and Physics, Charles University, Prague 150 06, Czech Republic; ⊥ Central Nervous System Diseases Research, Boehringer Ingelheim Pharma GmbH & Co. KG, Biberach an der Riß 88400, Germany; # School of Biological and Health Systems Engineering, Arizona State University, Tempe, Arizona 85281, United States; ¶ Macromolecular Chemistry, Department of Chemistry and Biology, University of Siegen, Siegen 57076, Germany; ∇ LiST-Life Sciences Technology, Danube Private University, Wiener, Neustadt 2700, Austria

## Abstract

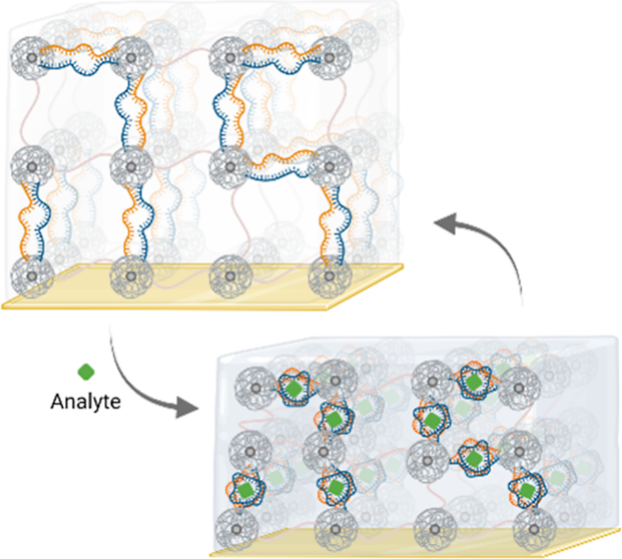

Continuous in vivo monitoring of small molecule biomarkers requires biosensors with reversibility, sensitivity in physiologically relevant ranges, and biological stability. Leveraging the real-time, label-free detection capability of surface plasmon resonance (SPR) technology, a molecularly responsive hydrogel film is introduced to enhance small molecule sensitivity. This advanced biosensing platform utilizes split-aptamer-cross-linked hydrogels (aptagels) engineered using 8-arm poly(ethylene glycol) macromers, capable of directly and reversibly detecting vancomycin. Investigation through SPR and optical waveguide mode, along with quartz crystal microbalance with dissipation (QCM-D) monitoring, reveals that the reversible formation of analyte-induced ternary molecular complexes leads to aptagel contraction and significant refractive index changes. Optimization of aptamer cross-link distribution and complementarity of split-aptamer pairs maximizes conformational changes of the aptagel, demonstrating a detection limit of 160–250 nM for vancomycin (6–9 fold improvement over monolayer counterpart) with a broad linear sensing range up to 1 mM. The aptagel maintains stability over 24 h in blood serum and 5 weeks in diluted blood plasma (mimicking interstitial fluid). This structurally responsive aptagel platform with superior stability and sensitivity offers promising avenues for continuous in vivo monitoring of small molecules.

## Introduction

Continuous in vivo monitoring of key analytes such as therapeutic drugs, glucose, proteins, and metabolites would enable improved real-time analysis of physiological changes and disease progression, fundamentally accelerating drug development and optimization of therapeutic strategies.^1,2^ Despite extensive research, glucose remains the only analyte successfully monitored continuously in vivo for extended periods,^3,4^ underscoring the complexities of developing robust biosensors for diverse physiological settings.^5^ While current continuous glucose monitoring systems have made significant strides forward, attempts to measure other biomarkers face limitations in achieving specific and sensitive detection beyond subcutaneous environments.^6,7^ To enable chronic monitoring across various biological environments, there is a pressing need for sensing platforms that are more durable, biocompatible, selective, and sensitive.

Hydrogels have emerged as promising materials to address the needs for durability and biocompatibility, offering tunable mechanochemical properties that mimic tissues.^8−10^ While hydrogels have shown success in monitoring physiological changes through stimuli-responsive systems sensitive to temperature,^11−13^ pH,^14−16^ and/or ionic strength,^17,18^ biomolecular detection requires typically more complex design requirements. These particularly include the need for incorporating highly specific molecular recognition elements for detection of low-concentration analytes, and effective signal transduction mechanisms.^19−21^ Aptamers, short oligonucleotides selected for specific target binding, have emerged as versatile recognition elements due to their high affinity, specificity, and sequence tunability.^22,23^ Aptamer-functionalized hydrogels harness this engineering flexibility within a responsive polymer network, enabling efficient transduction of molecular recognition events into detectable signals.^24,25^

Numerous research groups have explored aptamer-hydrogel composites for analyte detection, utilizing various functionalization methods and detection mechanisms based on the interplay between the hydrogel network, aptamers, and analytes. The simplest functionalization involves tethering full aptamer sequences within the hydrogel matrix, which allows the association of target analyte capture to local refractive index changes, similar to direct binding to immobilized antibodies,^26^ and is implementable in surface plasmon resonance (SPR) biosensors. For detecting low abundance and low molecular weight analytes, techniques derived from SPR for increased detection sensitivity, such as hydrogel optical waveguide spectroscopy (HOWS)^27,28^ or optical probing with long-range surface plasmons,^29^ were demonstrated. To amplify the response from bioreceptor-functionalized hydrogels by inducing physical property changes, such as swelling/collapsing or phase transitions, more complex aptamer-hydrogel designs were pursued. These approaches commonly utilize the principle of strand displacements caused by analyte binding to aptamer ssDNA strands, causing the gel to either swell^30−32^ or contract^33^ due to conformational changes of the polymer network. Other designs incorporate linker segments to promote more pronounced shifts, such as gel-to-sol transitions.^34^ However, these reported systems exhibit limited reversibility, requiring external stimuli or buffer changes for sensor regeneration, which is a critical limitation for enabling continuous biosensing applications.

Herein, we present an advanced biosensing platform based on split-aptamer-cross-linked hydrogel (aptagel) thin films probed by SPR, offering label-free detection at physiologically relevant concentrations while providing real-time molecular monitoring, with potential for miniaturization into implantable devices. We employed star-shaped, 8-arm poly(ethylene glycol)-norbornene (PEG-NB) macromers, known for their superior network homogeneity and cross-linking efficiency compared to linear polymers.^35,36^ Angular SPR with a wide-angle scan and quartz crystal microbalance with dissipation monitoring (QCM-D) were used to validate the proposed sensing mechanism by monitoring target analyte-induced changes in optical and viscoelastic properties, respectively, across various aptagel networks, designed with engineered cross-linking and split-aptamer pairs.

Following systematic aptagel optimization, including aptamer design, loading concentration, and cross-linking density, we analyzed the dose-dependent response for vancomycin as a model analyte to quantify key analytical parameters such as limit of detection, linear dynamic range, reversibility, and interaction kinetics. These optimized sensors were benchmarked against the conventional monolayer architecture on SPR. Their performance in complex biological matrices were evaluated in horse blood serum and diluted rat blood plasma (as a mimetic of interstitial fluid), to assess specificity and long-term stability. This multimodal approach facilitated the rational design of molecularly responsive hydrogel sensors with enhanced optical transduction, offering potential for sensitive and specific molecular detection in challenging in vivo environments for stable continuous monitoring applications.

## Results and Discussion

### Molecularly Responsive Aptagel Design for SPR Biosensing

We present the development of aptagel biosensors with SPR refractometric readout for direct, sensitive, and reversible detection of a model drug analyte, vancomycin. The design of an effective aptagel transducer required the capacity to induce optically detectable refractive index variations due to mechanical changes upon low-molecular weight analyte binding, to efficiently incorporate bioreceptors within the hydrogel matrix, and to stably attach onto a gold sensor substrate. Consequently, our design investigation was structured in three key components: (1) selection of hydrogel composition, (2) aptamer incorporation strategies into hydrogel, and (3) aptagel preparation on gold sensor substrate for characterization by SPR and waveguide modes.

### Hydrogel Composition

**Figure 1:**
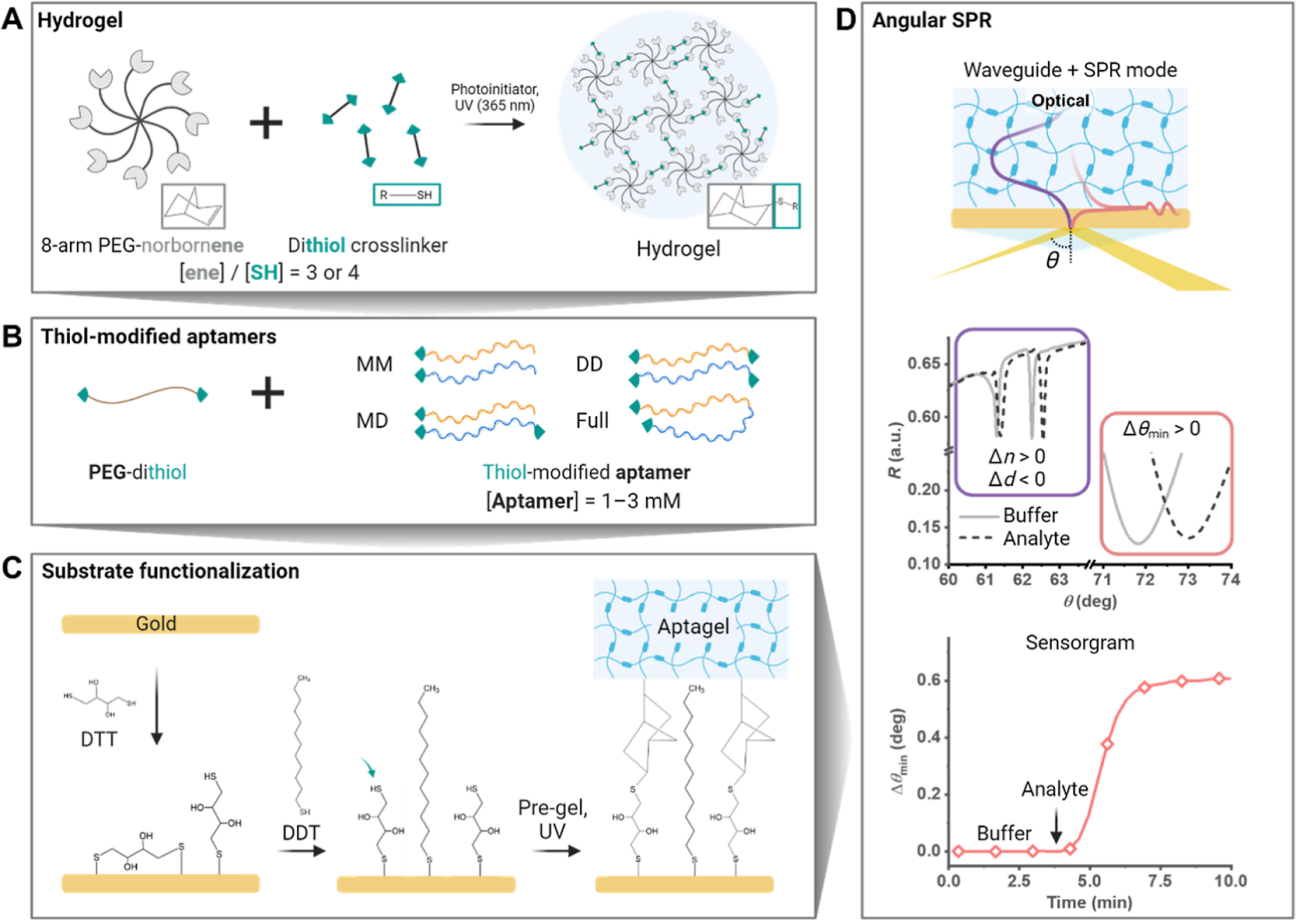
Experimental framework for investigating the aptagel on a gold surface. (A) The hydrogel was composed of star-shaped, 8-arm PEG-NB and dithiol cross-linker with a norbornene to thiol molar ratio ([ene]/[SH]) of 3 or 4, leaving free norbornenes available for covalent bonding with free thiols on the gold surface. (B) Thiol-modified aptamers were incorporated among the thiol-based cross-linker by varying the aptamer loading concentration from 1 to 3 mM. The following aptamer configurations were investigated: MM (monothiolated split-aptamer pairs), MD (one monothiolated and one dithiolated split-aptamer pair), DD (both split-aptamer pairs dithiolated), and Full (dithiolated, nonsplit, full parent aptamer sequence). (C) Gold surface prefunctionalization was performed by incubating the surface in dithiothreitol (DTT) and dodecanethiol (DDT) to promote a higher proportion of upright-oriented free thiols. Subsequently, the hydrogel precursor solution (pregel) was cast on the thiolated gold surface and exposed to UV radiation for 2 min to initiate the cross-linking of the aptagel network. (D) For optical analysis, angular SPR was utilized to determine the refractive index (*n*) and thickness (*d*) of the hydrogel film by examining the waveguide pattern (purple), while the surface plasmon resonance range (red) was used to investigate the minimum of SPR incident angle (θ_min_) shifts and study binding kinetics. Schematics are for illustrative purposes only and not drawn to scale.

We designed the hydrogel composition with the goals of achieving a tunable hydrogel network, efficient incorporation of the aptamers, and robust attachment of the hydrogel films to the gold sensor surface. The star-shaped 8-arm PEG architecture was chosen as it holds several advantages over a linear polymer design. Star polymers, characterized by at least three macromolecular chains stemming from a central core, possess unique topological structures and physical/chemical attributes.^37^ First, the star-shaped architecture provides improved network homogeneity for better controllable gel network structure compared to linear polymers, resulting in improved predictability of mechanical behaviors.^35,36^ Second, this topology enhances cross-linking efficiency through higher number of reactive end-groups,^37^ resulting in high-density functional groups that improve stimuli-responsiveness and functional versatility, while facilitating stable surface attachment.^38−40^ Third, the reduced solution viscosity compared to linear counterparts of equivalent molecular weight, due to fewer arm entanglements,^41^ facilitates the production of thin, homogeneous hydrogel films with improved reproducibility. The norbornene group of 8-arm PEG-NB can efficiently undergo covalent thiol–ene coupling reaction with thiol groups, either present in the cross-linking agents or on the gold sensor surface (Figure 1A).^42^

We prepared a thin hydrogel film on a gold-coated sensor substrate by utilizing these combined properties. The properties of the hydrogel were tuned by adjusting the molar ratio between norbornene (ene) units and total amount of thiol (SH) groups stemming from dithiol cross-linkers (PEG-dithiol) as well as from thiolated aptamers, denoted [ene]/[SH], while maintaining a constant macromer PEG-NB concentration of 10 mM. At [ene]/[SH] below 1, free thiol groups in the hydrogel served as sites for direct covalent bonding to the gold surface. However, this approach required excessively high thiolated aptamer concentrations, leading to material waste and reduced reproducibility due to difunctional aptamers forming monovalent attachments to the hydrogel. Consequently, [ene]/[SH] exceeding 1 was determined optimal, providing free norbornene groups capable of forming covalent bonds with the thiolated gold surface. Initial investigations revealed that [ene]/[SH] below 2.5 yielded insufficient attachment strength on the gold surface, while [ene]/[SH] above 4.5 exhibited weak mechanical properties due to decreased cross-linking density. Therefore, [ene]/[SH] of 3 and 4 were chosen to ensure robust attachment to gold substrate and appropriate hydrogel mechanical characteristics for sensing under microfluidics.

### Aptamer Integration

Previously, we designed and optimized a series of vancomycin-binding split-aptamer pairs for surface-based sandwich assay with SPR readout, demonstrating that its specificity and full reversibility allow for real-time monitoring of vancomycin under physiological conditions (such as at 37 °C and relevant ionic content).^43^ We incorporated these bioreceptors into the hydrogel matrix via thiol moieties, while the backbone of hydrogel structure was mainly maintained by PEG-dithiol cross-linkers (Figure 1B). Initial screening and optimization utilized the split-aptamer pair denoted P27, where the original aptamer sequence was split at base 27, and the derivative pairs were subsequently evaluated. We refer to our aptamer-functionalized hydrogel construct as “aptagel.”

Notably, P27 split-aptamer pairs exhibit temperature-dependent hybridization properties (Figure S1): at ambient temperature (∼25 °C), pairs spontaneously anneal, while at physiological temperature (37 °C), hybridization is minimized. We strategically exploited this characteristic by performing photo-cross-linking of the pregel solution at room temperature, enabling self-assembly of split-aptamer pairs prior to cross-linking. This approach created a molecular templating effect that facilitated spatial prealignment of split-aptamer pairs in the hydrogel matrix, establishing a well-defined molecular architecture rather than a completely randomized network.

The formation of our aptagel relied on thiol-norbornene click chemistry, which offered several advantages for incorporating functional oligonucleotide sequences. This UV-initiated reaction proceeds via a radical-mediated mechanism between the thiol-modified aptamer segments and norbornene groups on the 8-arm PEG macromers. The reaction exhibits rapid kinetics, high efficiency, and proceeds without byproducts under mild conditions (PBS buffer, pH 7.4, 25 °C), making it ideal for biomolecule integration. Importantly, the stoichiometric control afforded by this orthogonal chemistry enabled precise tuning of cross-linking density through adjustment of the [ene]/[SH].

The molar ratio of PEG-dithiol to thiolated aptamers ranged from approximately 2 to 12, with aptamer concentrations varying between 1 and 3 mM, the upper limit being constrained by the practically available oligonucleotide stock concentrations. Various thiolated aptamer configurations were investigated, encompassing monothiolated split-aptamer pairs (MM), a combination of monothiolated and dithiolated split-aptamer pairs (MD), dithiolated split-aptamer pairs (DD), and the dithiolated full parent aptamer sequence (Full).

### Aptagel Preparation for SPR Biosensing

The aptagel formulation contained excess norbornenes ([ene]/[SH] > 1) available for additional thiol–gold attachment. To achieve covalent bonding between aptagel and sensor substrate, the gold substrate was functionalized through sequential incubations with thiolated molecules (Figure 1C). First, the gold surface was incubated in a dithiothreitol (DTT) solution in ethanol for 5 h, followed by a brief 5 min incubation in a dodecanethiol (DDT) solution in ethanol. This protocol was shown to maximize the number of free thiol groups available for reaction with the norbornene groups.^44^ Next, the hydrogel precursor (pregel) solution was drop cast onto the thiolated gold surface and a glass slide was then gently pressed on the top. Finally, the sandwiched assembly was UV photo-cross-linked to form a thin hydrogel film that was covalently attached to the gold surface. Further testing revealed that UV exposure times up to 10 min in our experimental setup had no detectable impact on aptamer functionality or aptagel performance (Figure S2).

The thin aptagel layers prepared on gold-coated sensor chips were studied by angular SPR across a wide range of incident angles (θ) from 40 to 78° to simultaneously determine the refractive index (*n*) and thickness (*d*). This was possible by recording reflectivity changes associated with the excitation of optical waveguide modes traveling inside the thin aptagel layer (purple), while also detecting local refractive index changes occurring at the proximity to gold surface by the SPR (red) (Figure 1D). The resonant excitation of optical waveguide modes manifests itself as a series of narrow discrete resonance dips in the angular reflectivity spectrum, and they are highly sensitive to the optical and physical properties of thin hydrogel film. We determined its *n* and *d* by fitting the measured angular spectrum with a multilayer Fresnel reflectivity model.^45^ This approach is particularly well-suited for analyzing several microns thick hydrogel films, as the combination of waveguide and SPR modes provides insights into the film density gradients and analyte–aptamer interaction kinetics throughout the hydrogel matrix.

### Optical and Viscoelastic Properties of the Aptagel

#### Aptagel as Plasmonic Biosensor Architecture

**Figure 2:**
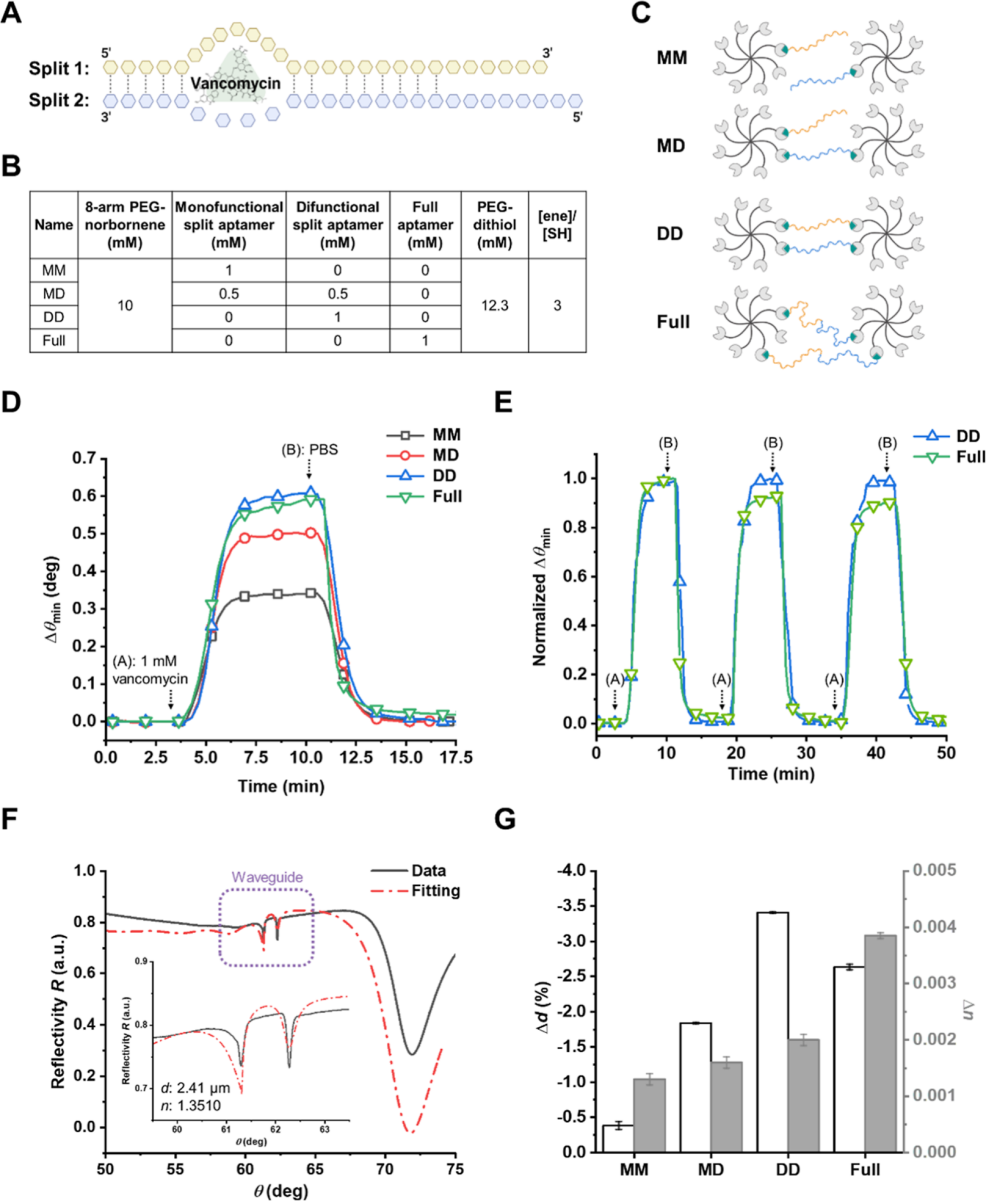
Effect of aptamer cross-linking configuration on vancomycin sensing observed by waveguide and SPR mode. (A) Structure of the P27 split-aptamer pair used in this study. Dotted lines indicate complementary bases (stem), with the vancomycin binding pocket located around the internal bulge. (B) Schematic illustration of different aptamer cross-linking configurations, based on thiol functional group allocations. (C) Composition of various aptagel formulations, detailing the ratios of key components. (D) SPR sensorgrams depicting vancomycin-induced response for different cross-linking configurations. (E) Repeated injection of 1 mM vancomycin comparing the reversibility and reproducibility of DD and Full aptagel. Sensorgrams were normalized between the first data point before injection (representing the baseline) and the highest data point of the data set. (F) Representative case of experimental data fitting to determine the refractive index and optical thickness of the hydrogel. The inset provides a magnified view of the waveguide mode from the full spectrum, marked by a purple box. (G) The changes in the thickness (Δ*d* %) and refractive index (Δ*n*) were calculated to compare the effect of 1 mM vancomycin binding on different cross-linking configurations. Error bars represent standard deviations (*n* = 3).

The primarily used split-aptamer pair in this study, P27, consisted of separate segments 1 and 2, whereas these segments were connected in the full-length aptamer sequence (Figure 2A). The binding site for the target analyte, vancomycin, was presumably located around the inner bulge of the aptamer,^43,46^ hence the split site was strategically positioned to ensure the binding pocket would be preserved. To initially assess the aptagel on vancomycin detection, [ene]/[SH] was fixed at 3, corresponding to a concentration of 10 mM for the PEG-NB and 12.3 mM for the PEG-dithiol cross-linkers (Figure 2B).

Four different aptamer configurations were investigated, as schematically represented in Figure 2C, involving varying tethering methods for the split-aptamer pairs or the full-length aptamer. The schematic provides an idealized representation of cross-linking in the hydrogel network, simplifying the actual complexity. It is important to note that this depiction does not capture all possible configurations of aptamer attachment such as intramolecular cross-linking within a single PEG-NB macromer. The aptamer concentration was then maintained at a constant 1 mM across all hydrogel samples. The difference in cross-linking density between the dithiolated (DD) and monothiolated (MM) aptagels was less than 8% (13.3 and 12.3 mM of dithiol linkers, respectively). By keeping the mechanical properties of the hydrogel matrix relatively uniform across the different aptamer configurations, this experimental design enabled the direct evaluation of how the varying aptamer incorporation strategies impact the aptagel response and sensitivity toward the vancomycin target, without confounding factors related to substantial changes in the underlying hydrogel network structure and mechanics.

After preparing thin hydrogel films on the SPR gold chips, the system was equilibrated at 37 °C and maintained throughout the experiment to mimic physiological conditions. Once thermal equilibrium was reached, a continuous flow of 50 μL/min was sustained until a stable baseline of the sensor response was achieved, ensuring a steady-state environment for accurate kinetics and providing consistent hydrodynamic conditions throughout the experimental duration. The reflectivity was monitored across a wide angular range with the θ_min_ of SPR used to generate the sensorgram.

The extent of θ_min_ shift (Δθ_min_), obtained by subtracting the baseline value, was presented to compare the response of aptamer-functionalized hydrogels to the analyte (Figure 2D). After stabilization, 1 mM vancomycin was injected into the measurement chamber (point (A) at 3 min), and time-dependent vancomycin binding kinetics were monitored. The monofunctionalized MM aptagel, with split-aptamer pairs attached to the hydrogel matrix via a single thiol group at one end, exhibited the smallest binding response compared to other aptamer configurations. The hybrid MD aptagel, incorporating a combination of monofunctionalized and difunctionalized split-aptamer pairs, showed an improved binding response compared to the MM aptagel but lower than the fully difunctionalized DD aptagel. These results suggest that difunctionalized aptamer configurations offer the best performance in terms of binding affinity and sensitivity toward vancomycin. Importantly, after buffer rinsing (point (B)), rapid dissociation kinetics are observed for all configurations, indicating a reversible interaction between the aptamers and vancomycin.

To assess the reversibility and reproducibility of the best-performing DD and Full aptagels, repetitive vancomycin injection sensing was conducted. Figure 2E reveals that full aptagel exhibited partial irreversible binding and low reproducibility, retaining only 90% of the initial signal after the third binding cycle. Conversely, DD configuration demonstrated fully reversible binding and high reproducibility over repeated vancomycin injections. Notably, DD aptagel exhibited excellent temporal resolution, with both association and dissociation kinetics occurring within a rapid 3 min time frame, highlighting the potential for real-time monitoring applications. These findings suggest that DD configuration, the engineered split-aptamer pairs with both terminal ends securely tethered to the 8-arm PEG-NB hydrogel, represents the most suitable design for achieving the desired reversibility and consistency in target analyte sensing performance.

For a more thorough examination of the mechanism, the *n* and *d* of aptagels were determined by analyzing the waveguide mode with a Fresnel reflectivity-based model (Figure 2F).^45^ The aptagel films exhibited consistent optical and physical properties across all formulations used in this study, with a *n* of 1.3486 ± 0.0017 and *d* of 2.59 ± 0.72 μm. All films demonstrated a swelling ratio of approximately 9, indicating a sufficiently open polymer network to facilitate rapid diffusion of target analytes regardless of compositional variations. The changes in the optical properties of aptagel induced by the presence of vancomycin are summarized in Figure 2G. The shift in refractive index (Δ*n*) was determined by directly subtracting the values before and after vancomycin introduction, and the change in thickness (Δ*d*) was calculated as a percentage change using the formula (*d*_after_ – *d*_before_)/*d*_before_*100.

A key property we succeeded in achieving through our aptagel design is that upon vancomycin interaction with the aptamers within the hydrogel matrix, the Δθ_min_ and Δ*n* increased, while the Δ*d* decreased. This indicates that the aptamer-analyte binding induces a physical change or actuation by altering the internal structure of the hydrogel. We hypothesize that the binding of vancomycin to the aptamers causes them to adopt a more compact conformation, leading to a collective contraction of polymer chains in the hydrogel network. Consequently, this contraction results in a decrease in the overall thickness of the hydrogel film. The extent of this actuation is likely dependent on the specific aptamer configuration, with the magnitude of the response varying accordingly. For instance, the MM configuration exhibited minimal changes in both thickness and refractive index upon vancomycin binding, while the MD displayed a slightly enhanced response compared to MM (Figure 2G). Remarkably, the DD configuration showed the most significant Δθ_min_ and Δ*d*. These findings emphasize the crucial role of the aptamer immobilization configuration in controlling the responsiveness to the target analyte, with the DD aptagel demonstrating the most promising performance in terms of hydrogel actuation, sensitivity, and reversibility.

#### QCM-D Insights Revealing Aptagel Sensing Mechanism

**Figure 3:**
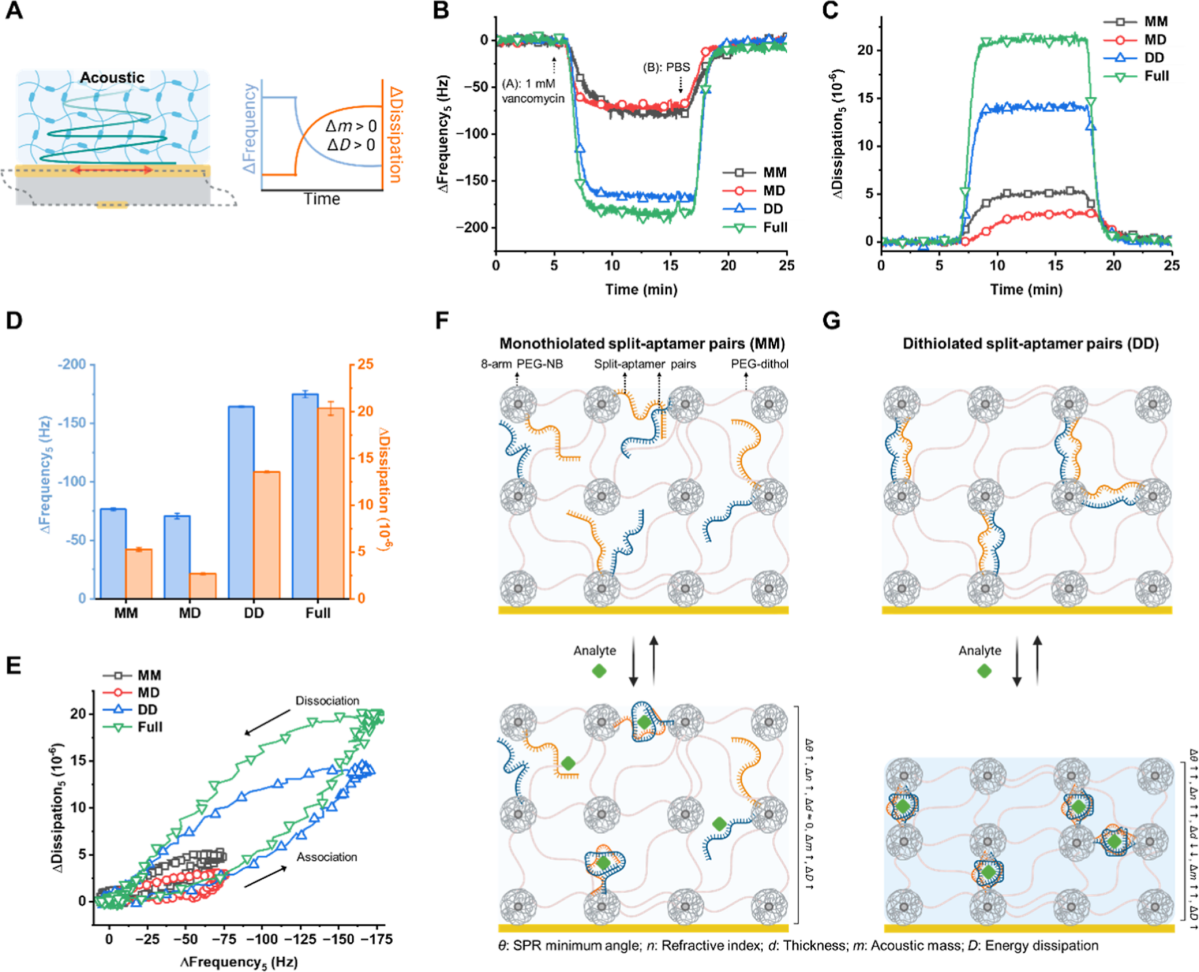
Acoustic characterization of aptagels to investigate the influence of aptamer cross-linking configuration on viscoelastic properties. (A) Quartz crystal microbalance with dissipation monitoring (QCM-D) was employed to assess the wet acoustic mass (*m*) via changes in resonance frequency (Δ*f*) and energy dissipation (Δ*D*), enabling the characterization of the viscoelastic properties of the hydrogel film during analyte–aptamer interactions. (B,C) Representative QCM-D frequency and energy dissipation shifts observed during interaction of aptagel and 1 mM vancomycin. (D) Maximum shifts in QCM-D resonance frequency and energy dissipation (5th overtone). Error bars represent standard deviations (*n* = 3). (E) Time-independent Δ*f*–Δ*D* plots constructed to provide insights into the viscoelastic contributions of the adsorbed species. (F,G) Schematic illustrations depicting the proposed mechanism of dithiol and monothiol split-aptamer pairs upon analyte binding, leading to the observed optical and acoustic changes in the hydrogel layer.

To further investigate the mechanistic details of the aptagel and complement the optical analysis, quartz crystal microbalance with dissipation monitoring (QCM-D) was employed. This technique enables the study of hydrodynamically coupled mass changes and energy dissipation caused by the viscoelastic properties of the adsorbed material. QCM-D relies on the oscillation of a quartz crystal, which is sensitive to changes in water-coupled mass on the surface, as indicated by changes in the resonance frequency (Δ*f*) and the energy dissipation (Δ*D*). A decrease in Δ*f* indicates an increase in mass due to bound molecules, while an increase in Δ*D* suggests greater energy loss caused by the viscoelastic nature of the hydrogel and coupled analyte (Figure 3A). To maintain consistency across measurement techniques, the QCM-D experiments were conducted on the surface of a gold layer under identical conditions to those of the SPR measurements, including a temperature of 37 °C, a continuous flow rate of 50 μL/min, and baseline stabilization prior to analyte introduction. This approach enabled characterization of the viscoelastic properties of the hydrogel film during aptamer-analyte interactions, providing insights into the dynamic behavior of the aptagel during binding events.

The vancomycin binding-induced kinetics of the aptagel viscoelastic properties are illustrated in Figure 3B,C, which display the temporal changes in viscoelastic properties reflected by Δ*f* and Δ*D*, respectively, for various aptagel configurations. Upon introduction of 1 mM vancomycin at ∼5 min, its interaction with all aptagels was manifested as a sharp decrease in resonance frequency, confirming mass increase due to vancomycin binding (Figure 3B). Correspondingly, Figure 3C shows the dissipation increase upon vancomycin introduction, likely due to increased molecular interactions and structural rearrangements within the gel layers. Both graphs demonstrate a return to baseline upon buffer (PBS) introduction at ∼20 min, highlighting the reversibility of the molecular binding and the stability of the aptagel layers.

The maximum Δ*f* and Δ*D* signals are displayed in Figure 3D. The acoustic mass shift observed from the Δ*f* generally showed a similar trend to the SPR results, particularly for the Δθ_min_ and Δ*n*. However, the MM aptagel exhibited a slightly higher coupled mass shift compared to MD, which could be further elucidated by the Δ*D* signal. The larger dissipation shift of MM aptagel compared to MD indicates that the amount of freely interacting vancomycin within the gel matrix was higher for the MM configuration. This is likely due to the greater flexibility and mobility of the MM aptamer segments, which are attached to the hydrogel matrix at only a single point. The freely moving, unattached end of the MM split-aptamers may not be optimally positioned or oriented for efficient ternary complex formation, reducing the binding affinity and sensitivity toward vancomycin. In contrast, the DD and Full aptagels showed similarly high amounts of vancomycin association, as evidenced by their comparable Δ*f*. However, the Δ*D* of DD aptagel was significantly smaller than that of Full, indicating that the dithiolated split-aptamer configuration induced relatively rigid, elastic binding interactions with vancomycin, in comparison to the more viscoelastic binding behavior observed for the Full configuration.

To analyze the dependence of analyte-mediated hydrogel structural transformations on the aptamer configuration, time-independent frequency-dissipation (*f*–*D*) curves, which describe changes in the properties of the adsorbed layer, are presented in Figure 3E. Specifically, *f*–*D* curves provide insights into the relative conformational changes occurring throughout the association and dissociation phases^47^ involving vancomycin binding. The area enclosed within the *f*–*D* curve serves as a qualitative indicator of the extent of change in the adsorbed film properties, with a larger enclosed area corresponding to more extensive conformational changes in the film characteristics^48^ due to vancomycin-aptamer interactions.

For example, the Full aptagel was most affected by structural transformations upon vancomycin binding, likely due to conformational folding of the elongated, flexible aptamer strand in response to target analyte interaction—a well-documented phenomenon in electrochemical aptamer-based (EAB) sensors.^49,50^ This is also indicated by the steeper slope of the Full aptagel compared to DD, suggesting a more dissipative, viscoelastic binding behavior for the full-sequence aptamer. When interacting with a similar amount of vancomycin, the Full configuration induces higher energy losses compared to DD, with these effects being particularly pronounced during the dissociation phase rather than the association phase. Similarly, the MD configuration exhibited a more rigid adsorbed layer and reduced conformational freedom compared to the MM. As the MM split-aptamer pairs are tethered to the hydrogel matrix at only a single end, this configuration showed the larger dissipation shift than MD when exposed to a comparable amount of vancomycin.

Given the different sensing penetration depths of the techniques employed, a suggested mechanism observed from angular SPR (sensing penetration of SPR and waveguide mode ranging up to 200 nm^51^ and 10 μm,^52^ respectively) and QCM-D (sensing depth of 300 nm^53^) is schematically described in Figure 3F,G. The comparison between the MM (Figure 3F) and DD (Figure 3G) designs highlights how the aptamer functionalization strategy can profoundly impact the binding-induced changes and sensitivity toward the analyte. The secure dual-point attachment of the DD configuration, with the split-aptamer pairs tethered at both ends to the hydrogel matrix, appears to be a crucial factor contributing to its superior binding-induced response compared to the more loosely tethered MM configuration. In contrast, the single-point attachment of the MM split-aptamers allows greater flexibility and mobility, resulting in a more dissipative, viscoelastic binding behavior and reduced binding affinity toward vancomycin.

To further elucidate the molecular basis of these observations, we can draw upon our previous findings regarding the behavior of P27 split-aptamer pairs.^43^ The split position of P27 was optimized to balance adequate affinity and full reversibility by minimizing annealing at physiological temperature (37 °C). Notably, at 25 °C, below the melting temperature, these split pairs hybridize, a property we exploited during aptagel preparation. The pregel solution underwent photo-cross-linking at ambient temperature, wherein the annealing of split-aptamer pairs created a molecular template, facilitating their spatial alignment and subsequent efficient incorporation of the target analyte.

In the absence of the target molecule at 37 °C, a minimum fraction of split-aptamer chains hybridized, a behavior we hypothesize persists within the hydrogel matrix. This minimal interaction maintains the hydrogel in a swollen state as the split-aptamer pairs remain spatially separated. However, upon exposure to vancomycin, these segments coalesce to form a ternary complex (split 1-analyte-split 2). This association triggers a conformational change in the aptamers, leading to the observed contraction of the hydrogel network. The dual-functionalized nature of the DD configuration likely amplifies this effect, resulting in more pronounced conformational changes compared to other configurations. In summary, the comparative analysis of these aptamer configurations, facilitated by the complementary sensing depths of SPR, waveguide mode, and QCM-D techniques, provides valuable insights into the binding mechanisms and underscores the critical role of aptamer immobilization in dictating the efficiency of target capture within the hydrogel matrix and, hence, the sensor performance.

### Aptagel Optimization for Enhanced Vancomycin Monitoring

#### Tuning Aptagel Formulation

**Figure 4:**
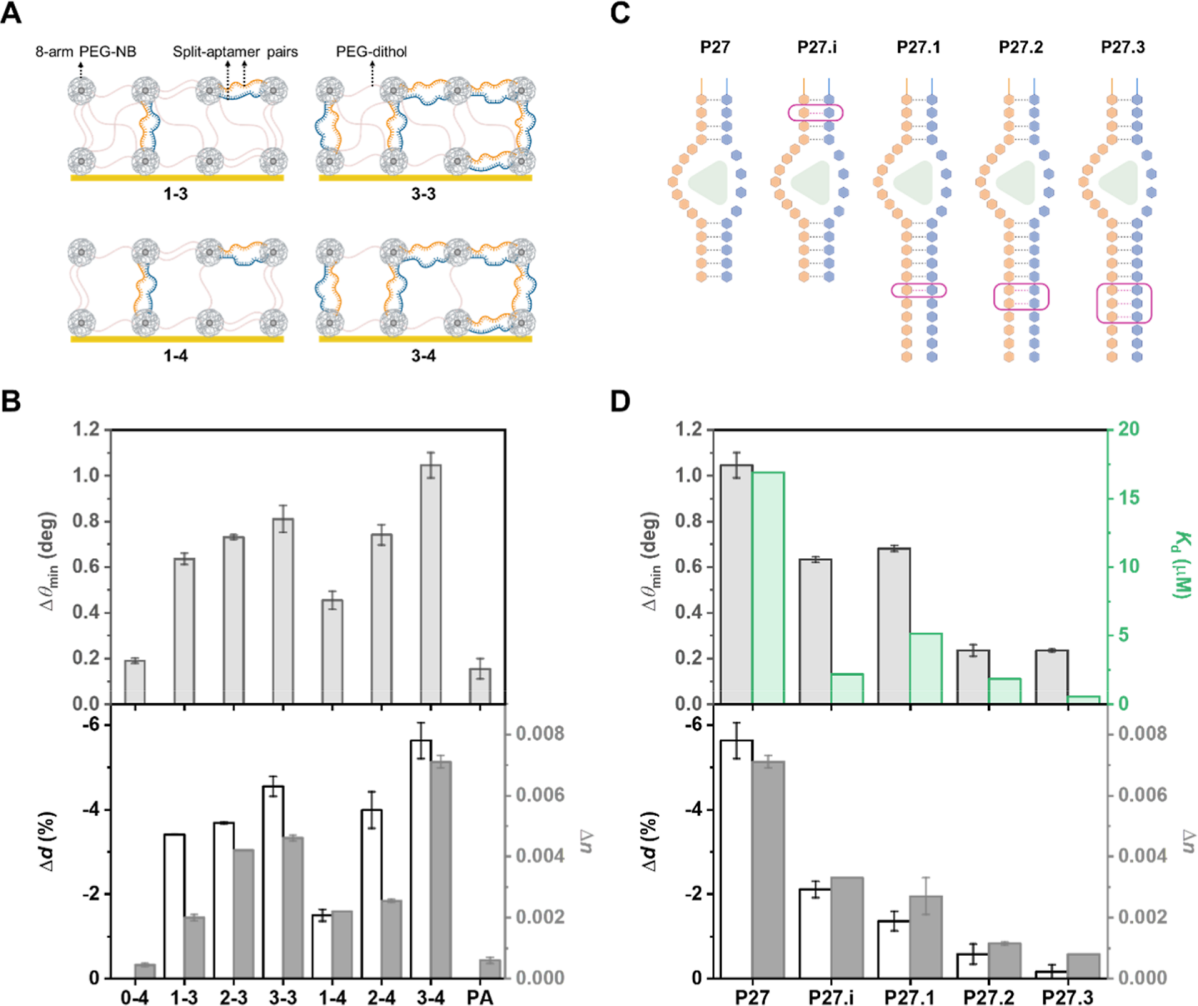
Effect of aptagel formulation and split-aptamer complementarity on sensing performance. (A) Schematic representation of aptagel formulations with varying aptamer proportions and cross-linking densities. *x*–*y*: *x* represents the aptamer loading concentration (mM), while *y* denotes the cross-linking density ([ene]/[SH]). A larger *y* value indicates a lower [SH], resulting in a lower cross-linking density. (B) Quantitative comparison of aptagel responses to 1 mM vancomycin. The graph shows Δθ_min_ (gray), Δ*d* (red), and Δ*n* (blue) for different aptagel formulations, including negative controls (0–4: blank hydrogels without aptamers; PA: hydrogels with nonspecific poly(A) sequence). (C) Structural representation of the P27 and its modified versions (P27.i to P27.3). Pink boxes highlight additional complementary bases introduced in each variant. (D) Comparison of sensing performance for engineered split-aptamer pairs. A positive correlation was observed between the apparent dissociation constant (*K*_d_, inversely related to binding affinity) of the split 1-analyte-split 2 complex from monolayer assay^43^ and Δθ_min_, suggesting that the complementarity and/or secondary structure of P27 represents an optimal balance for aptagel functionality. Error bars represent standard deviations (*n* = 3).

As the dithiol modification of both split-aptamer pairs (DD configuration) has been identified as the most efficient design, the optimized hydrogel composition was evaluated with two key variations (Table S1). The first variation involved the aptamer loading concentration (identified with a descriptor *x*), ranging from 1 to 3 mM. The second variation was the [ene]/[SH] (identified with a descriptor *y*), set at values of 3 and 4 to control the cross-linking density, where a larger value indicates a lower concentration [SH], resulting in a lower cross-linking density. These variations were incorporated into the sample using a convention (*x*–*y*), as illustrated in Figure 4A. To validate the specificity of the aptamer-analyte interaction, negative controls were included in the study. These controls consisted of a hydrogel without aptamers (0–4) and a hydrogel functionalized with a nonspecific poly A sequence (PA3–4). The inclusion of these negative controls allows for the assessment of aptamer specificity to analyte and the elimination of potential false-positive responses.

The SPR responses for the various hydrogel compositions, screened with 1 mM vancomycin, are presented in Figure 4B. Generally, increases in aptamer loading concentration led to larger shifts in all measured parameters (e.g., 1 – *y* < 2 – *y* < 3 – *y*), attributable to higher aptamer density within the hydrogel matrix facilitating more efficient interactions with diffusing vancomycin molecules. Additionally, the magnitude of these responses was more pronounced at lower cross-linking densities. Specifically, the effect was more evident in *x* – 4 compared to *x* – 3, where the higher [ene]/[SH] in *x* – 4 results in fewer cross-links, creating a more flexible network. This suggests that a reduced density of hydrogel backbone (neutral PEG dithiol) provides increased conformational freedom for split-aptamers to coalesce upon vancomycin binding. While 1–4 performed less effectively than 1–3, the 2 mM aptamer containing aptagels (2–3 and 2–4) exhibited similar performance. At 3 mM aptamer concentration, 3–4 significantly outperformed 3–3, establishing it as the optimal formulation. These results indicate that the interplay between aptamer loading concentration and cross-linking density controlled by [ene]/[SH] is complex, with optimal performance achieved at higher aptamer concentrations and specific cross-linking densities. The negative controls, 0–4 and PA, showed negligible responses, confirming the specificity of the aptagel for vancomycin detection.

It is noteworthy that the aptamer content in the 3–4 formulation constitutes 30% of all cross-linking moieties (3 mM aptamers vs 7 mM PEG-dithiol). This exceptionally high aptamer density significantly surpasses what is typically achievable through conventional postpolymerization functionalization strategies in hydrogel systems for sensing.^27−29^ The integration of aptamers as structural components of the hydrogel network, rather than as pendant groups, enables this remarkably high functional density without compromising the structural integrity of hydrogel.^24^ This unique architectural feature of our aptagel system likely contributes substantially to its enhanced sensing performance and underscores the advantages of this design approach in biosensor development.

#### Impact of Engineered Split-Aptamer Derivatives on Vancomycin Monitoring

Building upon the optimized aptagel composition based on P27 aptamer sequence, we investigated a series of engineered split-aptamer pairs previously developed to improve affinity and limit of detection of P27 in monolayer SPR sensor surface.^43^ These derivatives included P27.i, featuring an increased complementarity in the upper stem, and P27.1, P27.2, and P27.3, incorporating 0-, 1-, 2-, and 3-base pair extensions in the lower stem, respectively (Table S2 and Figure 4C). In monolayer assays, the experimental setup involved immobilizing one end of split 1 on the sensor surface, while split 2 remained free-flowing in solution (Figure S3). Using this configuration, these modified split-aptamer pairs with increasing complementarities demonstrated improved affinity and sensitivity while preserving the reversibility.^43^ The enhanced performance in this setup can be attributed to the increased stability of the sandwich complex split 1-analyte-split 2, facilitated by the additional complementarity. Based on these observations in the monolayer format, we hypothesized that higher affinity between separated segments would similarly enhance analyte capture efficiency in the aptagel system, where both split segments are tethered within the hydrogel matrix.

Contrary to our expectations, all modifications designed to increase complementarity resulted in significantly diminished responses compared to the original P27 (Figure 4D). Strikingly, P27.3, the modification with the highest number of base pairs, exhibited a response indistinguishable from negative controls, reflecting only bulk changes caused by vancomycin diffusion with minimal thickness changes. To understand the underlying mechanism of this unexpected behavior, we revisited the dissociation constants (*K*_d_) obtained from our previous monolayer assays^43^ and juxtaposed them with the aptagel responses (Figure 4D, green). As complementarity between split-aptamer pairs increased, *K*_d_ decreased (indicating higher affinity), while the aptagel response diminished. This relationship can be attributed to a DD configuration, whereby increased complementarity enhances internal base pairing within the gel, creating a less flexible structure. While this may increase vancomycin-binding affinity in monolayer assays, it reduces the ability of aptagel to undergo conformational changes upon analyte capture, which in turn affects our measurement output. This relationship suggests that the complementarity of P27 represents an optimal balance for aptagel functionality in the DD configuration, achieving sufficient binding affinity without compromising responsiveness of the entire gel network.

The discrepancy between monolayer and aptagel results highlights the crucial influence of the 3D hydrogel environment on aptamer functionality. While increased complementarity enhances affinity in a 2D setting, it may constrain conformational flexibility in the 3D hydrogel, hindering structural changes necessary for sensing and hydrogel actuation. These findings align with previous observations that lower hydrogel cross-linking density enhances response. Both scenarios—reduced aptamer complementarity and decreased hydrogel cross-linking—promote structural dynamics/flexibility necessary for efficient sensing. This underscores a key principle in aptagel design: the need for maximum contrast between stability and flexibility at both molecular and matrix levels.

### Evaluation of Aptagel Continuous Biomonitoring Performance

#### Sensitivity and Detection Range of Aptagel Biosensor

**Figure 5:**
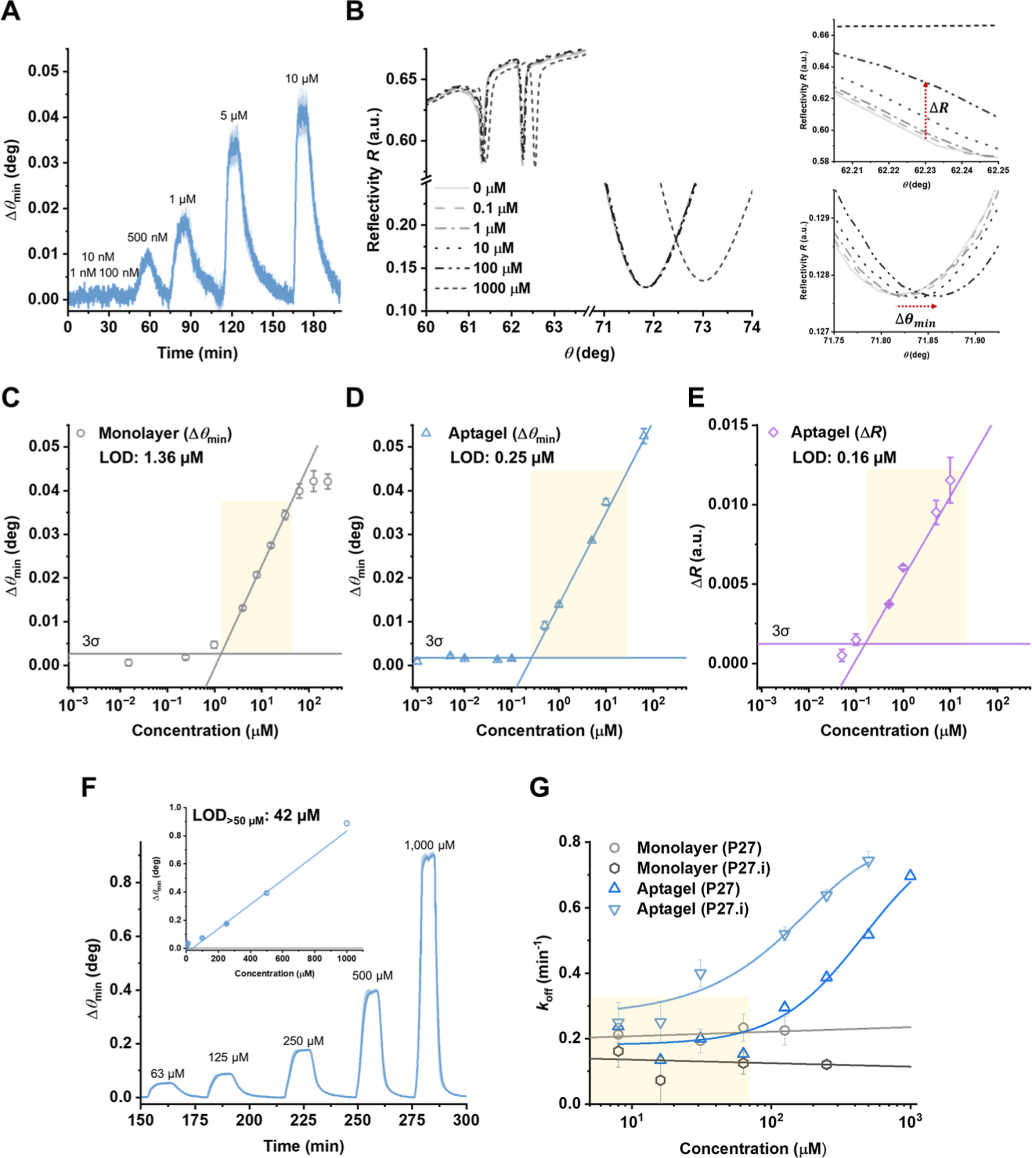
Sensitivity and detection range of aptagel biosensors. (A) Real-time sensorgrams showing responses to vancomycin (1 nM to 10 μM) in PBS buffer followed by rinsing step. Shaded areas represent standard deviation obtained from *n* = 3 measurements. (B) Full angular reflectivity (*R*) spectra of the aptagel at various vancomycin concentrations, demonstrating two tracking modes: (1) reflectivity changes (Δ*R*) of the waveguide and (2) Δθ_min_ of the SPR mode (insets). (C–E) Comparative analysis of monolayer and aptagel platforms. The limit of detection (LOD) was calculated from linear regression (yellow shaded region) intersecting at 3σ for each platform, where σ represents the standard deviation of the baseline signal. The aptagel shows improved LOD using both Δθ_min_ (0.25 μM) and Δ*R* (0.16 μM) compared to the monolayer (1.36 μM). (F) Aptagel response at higher concentrations, demonstrating linear relationship without saturation. Inset shows the linear calibration curve across the higher concentration range tested. (G) Dissociation rate (*k*_off_) as a function of vancomycin concentration. The yellow shaded region indicates the low concentration range where *k*_off_ remains relatively constant, corresponding to the linear response range for vancomycin detection in our aptagel sensor. Error bars represent standard deviations (*n* = 3).

The optimized 3–4 configuration with P27 aptagel was subjected to a dose–response analysis to evaluate its sensing performance. A wide range of vancomycin concentrations, spanning from 1 nM to 1 mM, were sequentially introduced into the measurement chamber, with intermediate rinsing steps to assess reversibility. The lower concentration range (1 nM to 10 μM) was initially examined, with the sensorgram tracking the Δθ_min_ as shown in Figure 5A. The aptagel sensor demonstrated full reversibility within 20 min and exhibited a detectable response from 500 nM.

Figure 5B presents the full angular reflectivity spectra for the aptagel at various vancomycin concentrations. The spectra reveal a more pronounced angular shift in the SPR mode (∼71.5 to 73°) compared to the waveguide mode (∼62.5 to 62.7°, observed from the first dip). It should be noted that due to the improved figure of merit (FOM), previous studies have demonstrated highly sensitive sensing by tracking the shift in reflectivity (*R*) in the waveguide modes using HOWS compared to conventional SPR sensors.^27,28^ The FOM relates to the sensor resolution and it is determined not only by sensitivity (defined as a ratio of the sensor response shift to changes in the refractive index of analyte) but also by the width of the resonant dip. Resolution typically exhibits an inverse relationship with the full width at half minimum (fwhm), which is significantly narrower in the optical waveguide mode compared to the SPR mode. In our system, the fwhm values were 0.094 and 3.39° for the waveguide and SPR modes, respectively, indicating the potential for a higher FOM in the optical waveguide mode. It is important to note that to accurately measure and compare this potential resolution gain, a finer angular step for the waveguide than for SPR mode would be needed, commensurate with the narrower fwhm. Without such an adjustment, the full resolution improvement potential of the higher FOM in waveguide mode may not be fully captured in comparative analyses.

To rigorously assess sensor performance, we conducted a comparative analysis of the limit of detection (LOD) between monolayer and aptagel assays. For the aptagel, we employed a dual-mode analysis, tracking both the Δθ_min_ and changes in reflectivity (Δ*R*) at a fixed angle in the waveguide mode, leveraging complementary information from distinct angular regions to achieve a comprehensive evaluation of the sensing capabilities of aptagel. The LOD was determined by linear regression analysis of the dose–response curve, as illustrated in Figure 5C–E. The LOD was defined as its intersection with three times the baseline standard deviation (3σ). The SPR (Δθ_min_) measurements revealed an LOD of 1.36 μM for the monolayer assay (Figure 5C), while the aptagel exhibited superior sensitivity with an LOD of 0.25 μM (Figure 5D). An additional dose–response analysis of the aptagel, using a pyramid-shaped concentration profile without intermediate washing steps to simulate real-life conditions, yielded consistent results with an identical LOD value (Figure S4). Notably, when monitored using the waveguide (Δ*R*) mode, the aptagel exhibited an order-of-magnitude lower LOD of 0.16 μM (Figure 5E) compared to monolayer assay. This improvement over the monolayer assay can be attributed to the 3D structure of aptagel, where split-aptamer pairs serve as cross-linkers, significantly increasing the density of binding sites and effective sensing volume.

The enhanced sensitivity of optical waveguide mode likely results from improved FOM and from its ability to probe the entire hydrogel volume, capturing cumulative binding events throughout the matrix.^27,28,54^ The analyte-induced shrinkage of hydrogel may further amplify the signal by increasing the local density of bound analytes within the sensing range. However, the modest improvement in sensitivity observed with Δ*R*, compared to previous reports,^27,28^ can be attributed to the homogeneous structure of our aptagel system. Unlike conventional approaches where hydrogels are surface-attached and subsequently modified, potentially resulting in heterogeneous receptor distribution,^27,28^ our method ensures uniform aptamer distribution by incorporating them into the pregel solution before rapid photo-cross-linking. This homogeneity likely facilitates rapid analyte diffusion, permitting and enhancing sensing with the SPR mode and explaining the less dramatic improvement in waveguide mode sensitivity often reported in less uniform systems. Given the comparable performance between SPR and waveguide modes, suggesting that volumetric sensing benefits are partially offset by efficient surface-based detection in our optimized aptagel, the SPR mode was selected for final sensor performance assessment.

We examined the response of aptagel across a wide range of analyte concentrations, revealing unique characteristics that provide insight into the mechanism and operational range of our sensor. At lower concentrations (<50 μM), the response was logarithmically proportional to concentration, mirroring monolayer systems. Above 50 μM, the response transitioned to a linear relationship up to 1 mM, an atypical extended range suggesting a unique sensing mechanism inherent to the 3D aptagel system (Figure 5F).

Next, we analyzed the concentration-dependent changes in the dissociation rate constant (*k*_off_) observed in the aptagel system (Figure 5G). In the monolayer system, P27 and P27.i exhibited *k*_off_ values of 0.2 and ∼0.16 min^–1^, respectively, aligning with our expectations of a slower rate for P27.i due to its higher affinity. Notably, these rates showed no dependency on analyte concentration, consistent with typical analyte-receptor binding kinetics for 1:1 interaction model, where dissociation is governed by the intrinsic stability of the complex rather than solution concentrations. In contrast, the aptagel system displayed more complex behavior. At vancomycin concentrations below ∼50 μM, *k*_off_ remained relatively constant at ∼0.2 min^–1^, corresponding to the logarithmic response region. However, above 100 μM, *k*_off_ increased markedly up to 0.7 min^–1^, coinciding with the transition to a linear response regime. This trend was observed for both P27 and P27.i, although P27.i generally exhibited higher *k*_off_ values, likely due to weaker interactions between the split-aptamer pairs and the analyte in aptagel.

This concentration-dependent increase in *k*_off_ may be attributed to several factors unique to the hydrogel environment. The 3D network allows for a higher capacity of analyte uptake, potentially leading to crowding effects and dynamic network interactions that could destabilize aptamer–analyte complexes.^55,56^ Examining the aptamer-to-analyte ratio offers further insight into this behavior. In our optimized DD configuration with a 3 mM concentration of split-aptamer pairs (1.5 mM functional binding units), the ratio ranges from 1500:1 at 1 μM vancomycin to 1.5:1 at 1 mM vancomycin. Notably, the transition in binding behavior occurs when this ratio drops below ∼15–30:1 (corresponding to 50–100 μM vancomycin), which aligns with our observed shift from constant *k*_off_ to increasing *k*_off_ values. This transition suggests a mechanistic shift: at high ratios, vancomycin molecules interact with relatively isolated binding sites, mimicking monolayer behavior with logarithmic response; as the ratio decreases, binding-induced local conformational changes begin to influence neighboring sites, causing deviation from conventional logarithmic relationships.

The cross-linking density of aptagel further modulates this effect, with lower densities providing greater conformational freedom for aptamer segments to form ternary complexes and undergo collective structural reorganization. This mechanical coupling through the hydrogel network explains why network contraction becomes more pronounced with increased binding site occupancy, potentially creating strain on existing complexes that facilitates dissociation and increases *k*_off_. Competitive binding and rapid rebinding within the gel matrix may become more pronounced at higher concentrations, while diffusion limitations could create heterogeneous concentration effects. Furthermore, the increased analyte load might induce subtle matrix deformations, altering the spatial arrangement and stability of binding sites. These combined effects, absent in the simpler 2D monolayer system, likely contribute to the observed increase in dissociation rates at higher vancomycin concentrations within the aptagel, highlighting the complex interplay between molecular interactions and the 3D hydrogel structure.

In summary, these results underscore the complex, concentration-dependent binding mechanisms in 3D hydrogel matrices and emphasize the need for a nuanced approach when translating aptamer designs from traditional assays to these more intricate sensing environments. The unique combination of factors in our aptagel system—including homogeneous structure, high aptamer density, and dynamic hydrogel reorganization—enables an extended linear range and sustained sensitivity at high analyte concentrations, distinguishing it from conventional biosensors. These observations provide insights into aptamer-analyte interactions within hydrogels and may inform future development of hydrogel-based sensing systems.

#### Specificity and Long-Term Stability Assessment in Biological Fluids

**Figure 6:**
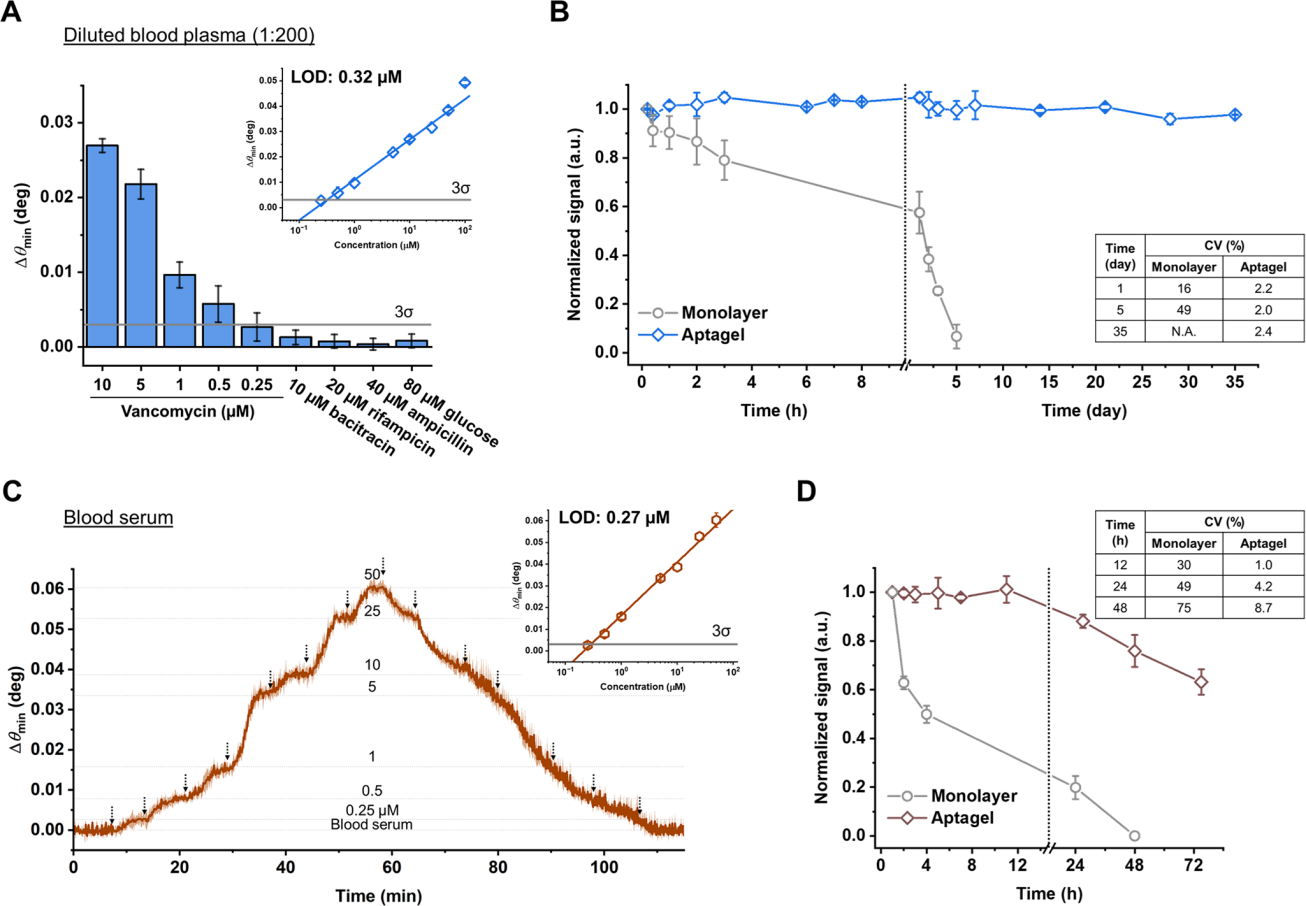
Sensor performance evaluation in diluted blood plasma and undiluted blood serum. (A) Dose–response and specificity in 200-fold diluted rat blood plasma, simulating interstitial fluid conditions. The LOD was 0.32 μM (inset). Common antibiotics and biomolecules with refractive indices matching that of 10 μM vancomycin elicited negligible responses (<3σ). (B) Long-term stability comparison between monolayer and aptagel sensors, assessed through repeated injections of 50 μM vancomycin over a 35 day period in diluted plasma. (C) Real-time sensorgram showing dose–response in undiluted horse blood serum, with concentrations ranging from 0.25 to 50 μM vancomycin. Inset shows the calibration curve with an LOD of 0.27 μM. Arrows indicate when each vancomycin concentration reached the microfluidic chamber. (D) Stability comparison between monolayer and aptagel sensors in undiluted horse blood serum over 3 days. Error bars represent standard deviations (*n* = 3).

With the aptagel system characterized, we evaluated its functionality in a complex, biologically relevant matrix. Biological fluids present additional challenges for biosensing due to their heterogeneous composition, their ability to degrade the sensor biointerface, and their tendency to mask the specific sensor response by nonspecific interactions. Our aptagel was designed to possess dual functionality: serving as a sensor transducer by self-containing receptor elements (aptamers) in a hydrogel matrix while simultaneously protecting the sensor surface from biological interferents. To assess the specificity and long-term stability of our aptagel in a physiologically relevant environment, we first employed a rat blood plasma solution diluted in assay buffer (1:200) to mimic the protein content of interstitial fluid.^43,57^ In this medium, the aptagel exhibited a clear dose-dependent response to vancomycin, with an LOD of 0.32 μM (Figure 6A), which is sufficiently low for detecting clinically relevant vancomycin concentrations.^29^

Crucially, we conducted specificity tests against a panel of potential interferents as negative controls mostly focused on potential clinical settings. Bacitracin served as our primary structural control due to its cyclic peptide structure similar to vancomycin’s core scaffold, comparable molecular weight, and multiple peptide bonds that could potentially interact with our sensing mechanism. Additional controls included rifampicin and ampicillin, antibiotics commonly administered alongside vancomycin in clinical settings,^58^ allowing us to evaluate potential interference in therapeutic scenarios. Glucose was included as a ubiquitous biological molecule to assess performance in complex biological matrices. The concentration of each interferent was chosen to match the refractive index of 10 μM vancomycin, based on their respective molecular weights (e.g., for glucose: 80 μM, 180Da ≈ 10 μM vancomycin, 1.5 kDa). All tested compounds elicited responses below the 3σ threshold of our vancomycin calibration curve, confirming the high specificity of our aptagel sensor in this complex matrix. These results demonstrate the robustness of our aptagel system in maintaining sensitive and specific detection capabilities even in the presence of potential interfering substances, underscoring its potential for practical applications in complex biological environments.

To assess long-term stability, we compared the aptagel to a conventional monolayer assay over an extended period of 35 days in diluted plasma at 37 °C (Figure 6B). At each time point, 50 μM vancomycin in respective medium was injected to obtain the response, with signals normalized to the initial readout, which was set to 1.0. The monolayer system showed rapid degradation, with signal intensity decreasing to ∼25% by the third day and approaching zero by the fifth day. In stark contrast, the aptagel biosensor maintained 100% of its initial performance for the first 3 weeks. To assess the long-term variability of the sensor, the coefficient of variation (CV) was calculated over the 5 week measurement period, yielding a value of 2.4%. This low CV indicates minimal fluctuation, demonstrating that the aptagel sensor maintained robust stability throughout the 5 week period without significant performance loss.

We further evaluated the sensor performance in undiluted horse blood serum to simulate more challenging physiological environments. Continuous monitoring of consecutive vancomycin injections at varying concentrations yielded a pyramid-shaped sensorgram (Figure 6C). The analytical performance of the aptagel sensor demonstrated remarkable consistency across different matrices, with minimal variation in the LOD between standard buffer (0.25 μM) and in blood serum (0.27 μM). Additional experiments conducted in 5-fold diluted human blood plasma resulted in an LOD of 0.68 μM (Figure S5). All measured LOD values remained in a favorable submicromolar range across different matrices, demonstrating robust sensor performance. The slightly higher LOD in human plasma likely reflects its characteristically greater heterogeneity, where protein aggregation was occasionally observed during measurements. Nevertheless, the maintenance of linear dose–response relationships in the calibration curves indicates that the fundamental binding kinetics and signal transduction mechanisms remain largely unperturbed by the complex composition of blood matrices, which contain numerous potentially interfering components.

Stability tests in blood serum showed no significant variation in sensor readout within a 12 h period with a CV of 2.5% (Figure 6D). At the 24 h mark, the sensor maintained 90% of its initial signal intensity with a CV of 4.2%, which remains within acceptable limits for continuous monitoring applications.^59^ However, by 48 h, signal intensity decreased to 75% of the initial readout, accompanied by an increased CV of 8.7%, indicating a decline in analytical reliability. In contrast, the monolayer assay exhibited rapid signal degradation, losing 50% of its original signal within 4 h, declining to ∼20% after overnight incubation, with a high CV of 49%. This represents a significant extension of sensor lifetime in serum from 4 to 72 h. We conclude that the aptagel sensor demonstrates superior stability in blood serum for at least 12 h, with reliability up to 24 h, significantly outperforming the monolayer assay. However, for extended periods beyond this, additional antifouling strategies may be necessary to maintain optimal performance.

This enhanced stability can be attributed to several factors. First, the protective nature of the 3D hydrogel matrix likely shields aptamer sequences from degradation and denaturation processes that typically occur in complex biological environments.^54^ This protective effect aligns with previous findings, where even a simple agarose coating extended the functional lifetime of electrochemical aptamer-based monolayer sensors for approximately 10 h.^60^ Second, the high density of cross-linked aptamers within the hydrogel structure may contribute to maintained functionality, even if some individual aptamers lose activity. Lastly, the semipermeability of hydrogel may exclude larger, potentially interfering molecules from the blood serum and may play a role in preserving sensor performance. These results suggest that the aptagel system can maintain detection capabilities in the presence of potential interfering substances over extended periods. The improved stability compared to monolayer systems indicates the potential advantages of this aptagel design for in vivo biosensor development.

## Conclusions

This study presents a comprehensive framework for incorporating split-aptamer pairs as cross-linkers within hydrogel networks, creating a novel “aptagel” platform for continuous biomarker monitoring. Through extensive analysis using angular SPR with wide-angle probing for both SPR and optical waveguide modes, complemented by QCM-D monitoring, we have developed and validated an aptagel-based biosensor with outstanding performance, including high sensitivity, while exhibiting excellent reversibility and reproducibility with long-term stability up to 5 weeks. This performance is attributed to the optimal formation of analyte-induced ternary molecular complexes within the hydrogel network, resulting in pronounced conformational changes and directly measurable network contraction without the need for additional labels. The developed platform represents a significant advancement in biosensing technology, offering potential for real-time, sensitive biomolecular detection and monitoring in various applications.

The strategic implementation of split-aptamer pairs, rather than full-sequence aptamers, proved essential for achieving rapid dissociation kinetics and complete reversibility in the sensing system. This design choice enables continuous monitoring capabilities with minimal hysteresis, a critical requirement for reliable real-time detection. Our experimental evidence demonstrates that alternative aptamer derivatives did not enhance aptagel functionality, confirming the optimal nature of our split-aptamer approach for dynamic biomolecular sensing applications where response time and reversibility are paramount. Optimization of the aptagel composition revealed that increased aptamer concentrations enhanced sensitivity, while cross-linking density modulated functionality. The aptagel system demonstrated 6-fold lower LOD (250 nM) compared to monolayer systems and nearly an order-of-magnitude lower LOD when probing with the SPR angular minima or optical waveguide modes, respectively, with a broad linear sensing range extending to 1 mM. Unexpectedly, engineered split-aptamer derivatives with increased complementarity, which showed improved performance in monolayer assays, exhibited impaired sensor response strength in the aptagel system, highlighting the complex interplay between aptamer structure and hydrogel-based sensing performance.

The aptagel sensor exhibited remarkable stability and specificity over 5 weeks in diluted blood plasma and 24 h in undiluted blood serum, addressing a key challenge in long-term biosensing within complex biological environments attributed to its antifouling property, homogeneous structure, high aptamer density, and dynamic hydrogel reorganization. The insights gained from this study will pave the way for developing advanced aptamer-based biosensors with improved performance and expanded applicability for in vivo biomolecular monitoring. Future work may focus on expanding to other analytes, coupling with more portable (e.g., optical fiber^61^) or sensitive (e.g., localized SPR^62^) sensor setups, and further optimizing aptamer sequences and hydrogel compositions based on the principles established in this study. For increasingly challenging in vivo environments such as blood vessels, additional antifouling strategies could be explored, including the incorporation of zwitterionic^63,64^ or engineered antifouling materials^65^ to mitigate biofouling and maintain sensor performance over extended periods. These advancements could potentially lead to even greater enhancements in sensor performance for continuous monitoring of a wide range of analytes clinically relevant across diverse healthcare and research applications.

## Experimental

### Materials and Reagents

8-arm PEG norbornene (10 kDa) was purchased from Creative PEGWorks. ssDNA aptamer sequences (Table S2) were synthesized by Integrated DNA Technologies with 5′ thiol modifier C6 S–S and/or 3′ thiol modifier C3 S–S for hydrogel matrix conjugation. All buffer reagents were purchased from Thermo Fisher Scientific as DNase/RNase-free stock solutions, including UltraPure distilled water, phosphate-buffered saline (PBS, 10×, pH 7.4), and magnesium chloride (1 M). HPLC grade ethanol and 1,4-dithiothreitol (DTT) 99% were acquired from Carl Roth GmbH. PEG-dithiol (MW: 3.4 kDa), lithium phenyl(2,4,6-trimethylbenzoyl)phosphinate (LAP), 1-dodecanthiol (DDT), tris(2-carboxyethyl)phosphine hydrochloride (TCEP), vancomycin hydrochloride, and lyophilized rat plasma were obtained from Sigma-Aldrich. 1-Ethyl-3-(3-(dimethylamino)propyl)carbodiimide hydrochloride (EDC) and *N*-hydroxysuccinimide (NHS), and neutravidin–biotin-binding protein were purchased from Thermo Fisher Scientific. Carboxyl and hydroxyl thiols (HS–(CH_2_)_*m*_–EG_6_–OCH_2_–COOH and HS–(CH_2_)_*m*_–EG_4_–OH) were purchased from ProChimia Surfaces, and methoxy-PEG-thiol (α-methoxy-ω-mercapto PEG) was purchased from Rapp Polymere GmbH. Horse serum was obtained from Gibco (cat. no. 16050130).

### Aptagel Preparation on Gold Substrate

The custom-made SPR gold sensor chip (dimensions: 20 × 12 × 0.5 mm) was purchased from LET Optomechanika Praha. The gold surface was functionalized by incubating the chip in a 50 mM solution of DTT in ethanol for 5 h, followed by a brief 5 min incubation in a 50 mM solution of DDT in ethanol. The surface was then thoroughly washed with water and ethanol, dried, and stored in a vacuum desiccator before further use. The hydrogel precursor solution was prepared by first mixing the respective amounts of DNA aptamers, PEG-dithiol, and TCEP (final concentration of 80 mM) in DNase-free water. The mixture was incubated at 37 °C for 2 h to reduce disulfide bonds to free thiol groups. Subsequently, 8-arm PEG norbornene and LAP (final concentration of 1 mM) were added to complete the pregel solution. A volume of 2 μL of this solution was drop-cast onto the treated gold surface, and a hydrophobic-treated glass slide was gently pressed onto the deposited solution. Finally, the sandwiched assembly was exposed to 365 nm UV light for 2 min to photo-cross-link the pregel solution and form a thin hydrogel film. The UV lamp was with 4 W UV output with 15 cm distance between the sample and the UV source, yielding UV intensity to be ∼1.4 mW/cm^2^, resulting in a total dose of 0.17 J/cm^2^ over 2 min.

### Monolayer Assay

The gold sensor chip was cleaned with ethanol, dried with nitrogen, and then exposed to a 1 mM solution of carboxyl and hydroxyl thiols (1:9 ratio) for at least 12 h to form a self-assembled monolayer. Subsequent functionalization and SPR measurements were conducted using an MP-SPR Navi 400 Kontio instrument. The sensor surface was first treated with methoxy-PEG-thiol for passivation, followed by activation of carboxyl groups with EDC/NHS. Neutravidin was then covalently attached to the surface, and unreacted sites were blocked with 1 M ethanolamine at pH 8, followed by immobilization of 1 μM 5′-biotinylated aptamer (split 1) in PBS (pH 7.4) for 20 min. Finally, excess biotin was used to occupy any remaining binding sites on the neutravidin.

### Angular Surface Plasmon Resonance (SPR)

The wide-angle SPR measurement was conducted using a dual-wavelength MP-SPR Navi 400 Kontio (BioNavis) instrument. The SPR was monitored at wavelengths of 670 and 785 nm, across an angular range of 40 to 78°, tracking the minimum SPR incident angle used to generate the sensorgram. Full spectra presented herein were reported with a wavelength of 670 nm. All measurements were performed at a temperature of 37 °C with a consistent flow rate of 50 μL/min, controlled by a peristaltic pump (Ismatec). The SPR gold sensor chip with the hydrogel film was inserted into the instrument, and the sample chamber was filled with the assay buffer (PBS containing 2 mM MgCl_2_). The system was allowed to equilibrate for at least 30 min to establish a stable baseline before the start of the experiment. After the baseline stabilization, analyte solutions were injected into the flow cell. To generate pyramid-shaped sensorgrams, the intermediate buffer washing step was omitted. Instead, vancomycin solutions of increasing concentrations were sequentially injected at intervals of ∼7 min, with a 4 min allowance for each solution to fully perfuse the microfluidic chamber. For long-term stability assessments in biological fluids, sensor chips were immersed in excess biological medium and maintained at 37 °C between experimental time points. Prior to each measurement, chips were transferred from the storage medium to the measurement chamber, where they were immediately exposed to the respective biological medium for analysis.

### Data Fitting and Analysis

The collected full spectra were fitted using the Fresnel reflectivity-based model implemented in WinSpall 3.01 software (Max-Planck Institute for Polymer Research, Germany).^45^ The initial optical parameters used in the software for the fitting were as follows: a laser source of 670 nm wavelength and a 4-layer system consisting of glass (*d* = 0, *n* = 1.52), gold (*d* = 48.94 nm, *n* = 0.2018), hydrogel (thickness and refractive index to be determined), and the aqueous solution (*d* = 0, *n* = 1.3337).^28^ The reference values for the glass and gold layers were obtained by fitting the bare gold sensor chip in deionized water, and maintained constant for all fittings to determine the *n* and *d* of hydrogel through iterative optimization of the full spectrum. The dissociation rate, *k*_off_, was calculated from the dissociation phase of the sensorgram by fitting the exponential one-phase decay function of the signal after the analyte solution is replaced with buffer. The coefficient of variation (CV) was calculated as the standard deviation (SD) divided by the mean, expressed as a percentage.

### Quartz Crystal Microbalance with Dissipation (QCM-D)

QCM-D measurements were conducted using a Q-Sense E4 instrument (Biolin Scientific) to monitor the kinetics of aptamer-functionalized hydrogel and analyte interactions. The changes in resonance frequency (Δ*f*) and energy dissipation (Δ*D*) of a quartz crystal were measured as a function of time. The quartz crystal was excited to generate the thickness shear mode at its fundamental resonance frequency of 5 MHz and at odd overtones (*n*: 1, 3, 5, 7, 9, and 11). All measurements were performed at 37 °C with a flow rate of 50 μL/min, controlled by a peristaltic pump (Ismatec). The gold-coated quartz crystal sensor (QSX 301, Biolin Scientific) was functionalized as described previously, and all data presented herein were collected at the fifth overtone. The system was allowed to equilibrate in the assay buffer for at least 30 min to establish a stable baseline. The collected data were analyzed using the QTools software (Biolin Scientific).

## Supporting Information Available

Aptagel formulations, DNA sequences of all aptamer variants, temperature-dependent hybridization, aptagel response by varying UV exposure times, schematic comparison of aptagel versus monolayer sensing platforms, continuous vancomycin detection sensorgram in assay buffer, sensorgram and stability comparison in diluted human blood plasma (PDF)
